# Book Notices

**Published:** 1894-12

**Authors:** 


### Book Notices.

Annual of the Universal Medical Sciences. A Yearly Report of the Progress of the General Sanitary Sciences Throughout the World. Edited by Charles E. Sajous, M. D., and seventy Associate Editors, assisted by over two hundred Corresponding Editors, Collaborators and Correspondents. Illustrated with Chromo lithographs, Engravings and Maps. Five volumes. Price, in cloth, $15. The F. A. Davis Company, Publishers, Philadelphia, New York, Chicago and London. Australian Agency: Melbourne, Victoria. 1894.

This is the seventh issue of this elaborate work. Like the preceding issues, it is issued in five beautiful volumes, and the work, as a whole, contains a report of the progress of medicine and surgery, in all their branches, throughout the world. With the aid of an able corps of associate editors, collaborators and correspondents representing every civilized country of the globe, Dr. Sajous has given us most of the practical results of investigations, observations and experiences of medical men during the past year.

No other work will enable a physician to so thoroughly familiarize himself with all that is new and useful in the practice of his profession. The medical writer can find no other work that will save him so much time and labor in research. With each issue, the work becomes nearer perfect, though one can scarcely see how the next is to be better than the present one.

The statement made by others that with the removal of the editor-in-chief to Paris the work would lose its distinctive American character, has not only not held good, but it is now as essentially American as ever before. More than two-thirds of the members of the editorial staff are Americans, among whom may be mentioned the following leading men in the profession: Prof. James T. Whittaker, Dr. E. S. McKee, Prof. S. Solis-Cohen, Prof. J. P. Crozer Griffith, Prof. Landon Carter Gray, Prof. James C. Wilson, Dr. Augustus A. Eshner, Prof. J. Lewis Smith, Prof. N. S. Davis, Prof. Frederick P. Henry, Prof. George H. Rohé, Prof. E. E. Montgomery, Prof. J. M. Baldy, Dr. Andrew F. Currier, Prof. Lewis S. Pilcher, Prof. J. McFadden Gaston, Dr. John H. Packard, Dr. W. T. Bull, Prof. Charles B. Kelsey, Dr. E. L. Keys, Prof. J. William White, Dr. W. A. Edwards, Prof. Lewis A. Sayre, Dr. Reginald H. Sayre, Prof. P. S. Connor, Prof. Lewis A. Stimson, Prof. Christian Fenger, Prof. Rudolph Matas, Prof. E. Laplace, Prof. L. McLane Tiffany, Prof. A. Van Harlingen, Prof. H. A. Hare, Dr. David Cerna, Dr. A. D. Rockwell, Dr. Simon Baruch, Prof. James O'Dwyer, Dr. Walter Wyman, Prof. W. Xavier Sudduth, and many others. From this list it will be seen that the work is distinctively American, and that the editorial staff embraces many of the brightest minds in the medical profession in America. We cheerfully commend the book, and believe that every doctor who desires to keep up with medical progress should have it. Much praise is due both the editor and the publishers. H.

---

Anomalies of Refraction and of the Muscles of the Eye. By Flavel B. Tiffany, M. D., Professor of Ophthalmology and Otology of the University Medical College of Kansas City, Mo.; Oculist and Aurist to All Saints' and German Hospitals; Oculist to the M. K. & T. R. R. Author's Edition. Three hundred and seven pages, twelve plates, and one hundred and eighty-seven illustrations. The Hudson-Kimberly Pub. Co., publishers, Kansas City, Mo.

The author treats of the anomalies of refraction in thirteen separate chapters, as follows: Chapter I, Reflection of Light. Chapter II, Refraction of Light. Chapter III, The Eye. Chapter IV, Emmetropia and Ametropia. Chapter V, The Accommodation of the Eye. Chapter VI, How to examine the Eye. Chapter VII, Myopia. Chapter VIII, Hypermetropia. Chapter IX, Astigmatism. Chapter X, Anisometropia, Aphakia, and Presbyopia. Chapter XI, Heterophoria. Chapter XII, Strabismus. Chapter XIII, Spectacles. An appendix in two parts, gives in part I, Synopsis of data gathered from the examination of 2040 school children of the public schools of Kansas City, Universities of Kansas and Missouri, State Normal and other district schools.

Part II, of appendix, describes a cabinet for holding various test types,—a prism pile—prism-measuring and lens-centering instrument—lens measurer, and several pages of test types.

The book also contains a full page portrait and a biographical sketch of Prof. von Helmholtz, Prof. Donders and Prof. Edmund Landolt.

The author has exercised great care, and has spent much time and labor in the preparation of this volume, and the result is a book of more than usual value to the student of diseases of the eye. The illustrations are excellent and profuse, and the mechanical work, paper, etc., is all that conld be desired. H.

---

A Manual of Instruction in the Principles of Prompt Aid to the Insured—Including a chapter on hygiene and the drill regulations for the hospital corps, U. S. A. Designed for military and civil use. By Alvah H. Doty, M. D., Major and Surgeon Ninth Regiment N. G. S. N. Y.; late Attending Surgeon to Bellevue Hospital Dispensary, New York. Second edition, revised and enlarged. Three hundred and four pages, profusely illustrated. Price, cloth, $1.50. D. Appleton & Co., Publishers, New York. 1894.

The possession by lay people of a knowledge of what to do to give temporary relief in cases of accidents and emergencies, would often result in the relief of much suffering, and sometimes in the saving of human life. That such information should be disseminated among non-medical men, is undisputed, but the strong probability that such knowledge would often be misused and the possessor of it make an effort to usurp the functions of a physician or surgeon to the detriment of the patient, should make us careful in our selection of books for such use. This one of Dr. Doty's seems to be as free from objections as any that could be selected, and the amount of information it contains is abundant and of mnch practical value. Not only the laity, but medical men as well, would gain valuable information by a careful perusal of it. The author has made a special effort to make it useful to the ambulance corps connected with the different military organizations, and the book is peculiarly adapted to their use. H.

---

ESSENTIALS OF THE PRACTICE OF PHARMACY. Arranged in the form of Questions and Answers. Prepared especially for Pharmaceutical Students. By Lucius E. Sayre, Ph. G., Professor of Pharmacy and Materia Medica, of the School of Pharmacy of the University of Kansas. Second edition, revised. Two hundred pages. Price, cloth, $1.00. Interleaved for notes, $1.25. W. B. Saunders, Publisher, 925 Walnut St., Philadelphia. 1894.

This is one of Saunders' popular question compends, and in this edition of the book the text has been revised so as to correspond with the United States Pharmacopœia of 1890. Some very important additions have been made in this edition, among which may be mentioned: An outline of Drug and Plant Analysis, Structural Formulæ of Organic Carbon Compounds used in Medicine, Pharmaceutical Testing of Inorganic Chemicals, and Problems in Allegation and Specific Gravity. The student of pharmacy will derive much benefit from the study of this volume, both in his preparation for attending a school of pharmacy, and in reviewing the course of study. H.

---

THE PHYSICIAN'S VISITING LIST (Lindsay & Blakiston's) FOR 1894.—Forty-fourth year of its publication. Price, in flexible Morocco cover, $1.00. P. Blakiston, Son & Co., Publishers, 1012 Walnut street, Philadelphia.

This is one of the most convenient, both in size and general arrangement, of all the visiting lists. The space for making entries of visits is ample; it also contains a number of pages for "Memoranda," "Address of patients and others," "Nurses' addresses," "Bills and accounts asked for," "Memoranda of wants," "Obstetric engagements," "Vaccination engagements," "Obstetric cases," "Record of deaths," "Cash account," etc. A large number of valuable tables, including "Posological table," "Dose Table," "List of New Remedies," "Incompatibility," "Poisons and Antidotes," "Disinfectants." and many others add much value to this visiting list. H.

---

THE WEEKLY MEDICAL REVIEW, POCKET REFERENCE BOOK AND VISITING LIST, PERPETUAL.—Price, in flexible Morocco cover, $1.00. J. H. Chambers & Co., Publishers, St. Louis, Mo.

This list is intended to supply the wants of the practitioner at the bed side. The blank pages are arranged in the usual form—

giving space to the weekly call list, obstetric calls, memoranda, etc., etc. The book also contains a nice calendar for 1895, and many valuable tables, among which may be mentioned—table of doses, doses of medicine for children, prediction of date of confinement, artificial respiration, care of galvanic batteries, clinical examination of urine, chemical examination of urine, diagnostic table of eruptic fevers, etc. The book is handsome and convenient. H.

